# A small number of daily pitches induces shoulder and elbow injuries among high school baseball pitchers: a prospective study

**DOI:** 10.1038/s41598-020-78957-0

**Published:** 2020-12-15

**Authors:** Hitoshi Shitara, Tsuyoshi Tajika, Takuro Kuboi, Tsuyoshi Ichinose, Tsuyoshi Sasaki, Noritaka Hamano, Takafumi Endo, Masataka Kamiyama, Atsushi Yamamoto, Tsutomu Kobayashi, Kenji Takagishi, Hirotaka Chikuda

**Affiliations:** Department of Orthopaedic Surgery, Gunma University Graduate School of Medicine, 3-39-22, Showa, Maebashi, Gunma 371-8511 Japan

**Keywords:** Health care, Medical research, Risk factors

## Abstract

Baseball players frequently injure their shoulders and elbows. Preseason risk factors for such injuries have been relatively well studied, but in-season risk factors are less known, and the relationship between the number of pitches and the incidence of such injuries in baseball pitchers of any level is unclear. Identifying the risk factors related to the number of daily pitches is particularly important to prevent baseball-related arm injuries among young pitchers. Thus, we prospectively investigated the relationship between the number of daily full-power pitches in high school baseball pitchers and the incidence of shoulder and elbow injuries. We observed that a small number of daily full-power pitches, < 30 pitches per day, in high school baseball pitchers, significantly increased the risk of shoulder and elbow injuries: these players had a 2.3-times greater risk of injuries and a 45-day earlier occurrence of injuries than those pitching ≥ 30 pitches per day. Although unexpected, this was plausible as continuous daily pitching is required to maintain physical condition in growing and maturing high school pitchers. These findings may form the basis for establishing guidelines regarding the appropriate number of daily pitches required to prevent shoulder and elbow injuries in high school baseball pitchers.

## Introduction

Baseball players commonly suffer from shoulder and elbow pain and injuries^1–3^. Recent systematic review^4^ for only previous prospective studies have demonstrated significant preseason risk factors for baseball-related arm injuries^5–13^. Among youth baseball players, Matsuura et al.^9^ have shown that significant risk factors for elbow injury were being a pitcher or a catcher^9^, being 9–11 years old^9^, and having a history of elbow pain^9^. Among high school baseball pitchers, prospective studies have identified a glenohumeral internal rotation deficit (GIRD)^10^, a greater difference in prone external rotation strength between the arms^10^, and supraspinatus weakness in the preseason^11^ as significant risk factors for elbow and shoulder injury. Among professional Major and Minor League baseball players, significant risk factors for elbow and shoulder injury were shoulder external rotation and elbow varus torque at peak external shoulder rotation during pitching^5^, high pitch velocity^6^, GIRD, shoulder external rotation insufficiency^15^, preseason total shoulder rotation deficit, preseason deficits in the supraspinatus, and prone external rotation strength^7^.

Thus, preseason risk factors have been relatively well studied. However, evidence for in-season risk factors, such as the number of pitches, innings, and external load (training and competition hours), is limited. Among youth baseball players, significant in-season risk factors for elbow injury were pitching > 100 innings in 1 year^14^ and training > 16 h per week^9^. In particular, there have been no reports on the relationship between the exact number of daily pitches and the incidence of shoulder and elbow injuries in baseball pitchers of any level. Nevertheless, evidence for risk factors related to the number of daily pitches is particularly important among in-season risk factors to prevent baseball-related arm injuries among young pitchers because the number of pitches in usual practice may be more than that in games. Therefore, as in-season risk factors, not only the number of pitches in the game but also the number of pitches in usual practice should be investigated.

Thus, this study prospectively investigated the relationship between the number of daily full-power pitches during usual practice and game in high school baseball pitchers and the incidence of shoulder and elbow injuries. “Full-power pitch” was defined as practical pitching from the pitcher’s mound toward the catcher either in a game or a usual practice, and it excluded the playing catch, such as warm-up pitching, toward the standing catcher from “a full-power pitch.”

## Results

### Injury

Of a total of 30 arm injuries, 12 shoulder injuries, 17 elbow injuries, and 1 shoulder and elbow injury occurred during the season. There was no situation such as an absence of games or daily practice because of continuous shoulder and elbow discomfort, before the injury occurrence.

### Receiver operating characteristics (ROC) analysis

To identify the cut-off point of numbers of daily full-power pitches for determining the incidence of shoulder and elbow injuries, ROC analysis was performed and subsequently revealed that the cut-off value of the average numbers of daily full-power pitches of incidence of shoulder and elbow injuries was 30 pitches per day (*P* = 0.33, area under the curve [AOC] = 0.57, Supplementary Figure S1).

### Baseline characteristics

On the basis of the cut-off value, participants were categorized into a group with a small number of full-power pitches (S group: < 30 pitches/day) and a group with a large number of full-power pitches (L group: ≥ 30 pitches/day). There were 38 and 52 pitchers in the S and L groups, respectively. The maximum number of pitches recorded in the L group was 115 pitches per day. In the preseason baseline assessment, no significant difference was observed between groups regarding baseball experience, height, weight, range of motion (ROM) of abducted internal rotation (ABIR) and horizontal adduction (HA) in the dominant shoulder, elbow flexion on the dominant side, prone external rotation (PER) and prone internal rotation (PIR) in the dominant shoulder, and PER and PIR ratios (Table 1). However, elbow extension ROM on the dominant side in the S group was significantly wider than that in the L group. Thus, in the preseason, the participants in the two groups had the same risk of experiencing shoulder and elbow injuries during the season, except for elbow extension ROM.

**Table 1:** Baseline characteristics of the study participants.

Baseline characteristics	S group (N = 38)		L group (N = 52)		P-value	
	Mean	SD	Mean	SD		
Baseball experience (years)	7.8	2.4	8.5	1.6	0.10	
Body height (cm)	172.1	5.8	173.3	4.6	0.29	
Body weight (kg)	67.3	7.1	68.5	6.4	0.42	
ABIR in dominant side (deg)	37.8	11.9	35.7	14.7	0.48	
HA in dominant side (deg)	30.1	14.3	26.3	10.0	0.18	
Elbow flexion in dominant side (deg)	144.1	4.8	143.6	5.0	0.61	
Elbow extension in dominant side (deg)	4.1	7.0	1.1	6.1	0.04	*
PER in dominant side (lb)	23.5	5.6	24.7	5.9	0.35	
PER ratio	1.0	0.2	1.0	0.2	0.87	
PIR in dominant side (lb)	25.2	7.4	26.7	8.6	0.40	
PIR ratio	1.0	0.2	1.0	0.2	0.66	

ABIR: range of motion (ROM) of 90° abducted internal rotation in the shoulder; HA, ROM of horizontal adduction in the shoulder; PER/PIR, muscle strength of prone external/internal rotation; ratio = strength in the dominant side/strength in the non-dominant side, *P < 0.05.

### Time-to-event analysis

**Figure 1:**
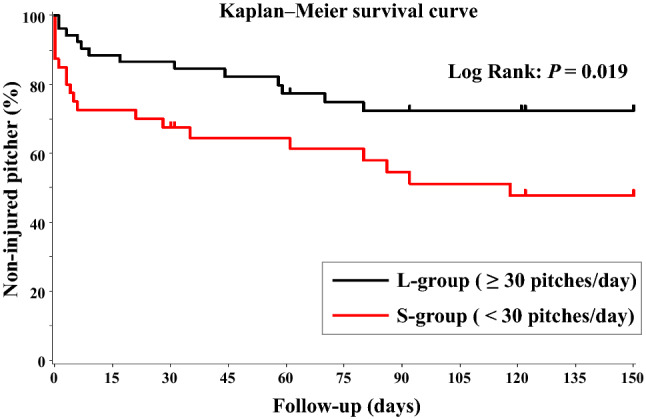
Kaplan–Meier survival curves. The median time to injury was 61.0 and 106.5 days in the S and L groups, respectively. A log-rank test showed that injury incidence was significantly higher in the S group than in the L group (*p* = 0.019).

The injury rates were 44.7% (n = 17) and 25.0% (n = 13) in the S and L groups, respectively. Similarly, the median time to injury was 61.0 and 106.5 days in the S and L groups, respectively (Fig. 1). This result suggested that a small number of daily pitches led to a 45-day earlier occurrence of injuries. Kaplan–Meier analysis yielded a hazard ratio (HR) of 2.269 in the S group compared to the L group (Table 2). A log-rank test showed that injury incidence was significantly higher in the S group than in the L group (*p* = 0.019; Fig. 1).

**Table 2:** Results of the Cox proportional hazards model analysis.

Group	Total	Incidence of injures		P-value
	N	N (%)	HR (95% CI)	
S group (< 30)	38	17 (44.7)	2.267 (1.120–4.597)	0.019
L group (≥ 30)	52	13 (25.0)	1	

*HR* hazard ratio, *CI* confidence interval.

### Post hoc power analysis

Post hoc power analysis showed that the statistical power of this study was 70.5%.

### Sensitivity analysis of the cut-off value

First, we set the shifted cut-off values by ± 5 (i.e. 25 and 35). Second, we performed the Kaplan–Meier analysis, Cox proportional hazard model analysis, and a log-rank test in the same manner as when a cut-off value of 30 pitches per day was used (Table 3). Finally, we compared the results using the shifted cut-off values to that using a cut-off value of 30 to confirm the appropriateness of 30 pitches per day as a cut-off value in this study.

**Table 3:** Results of the sensitivity analysis of the cut-off value.

Group	Total	Incidence of injures		P-value
	N	N (%)	HR (95% CI)	
**Cut-off value = 25**				
S-group (< 25)	26	12 (46.2)	2.186 (1.086–4.399)	0.024
L-group (≥ 25)	64	18 (28.1)	1	
**Cut-off value = 35**				
S-group (< 35)	47	19 (40.4)	1.898 (0.915–3.937)	0.078
L-group (≥ 35)	43	11 (25.6)	1	

*HR* hazard ratio, *CI* confidence interval.

A cut-off value of 30 pitches per day was superior in determining the incidence of throwing-related injury compared to the shifted cut-off values. Similarly, the sensitivity analysis supports the appropriateness of 30 pitches per day as the cut-off value in this study.

## Discussion

The most important finding of this study was that a small number of daily full-power pitches, < 30 pitches per day, in high school baseball pitchers significantly increased the risk of shoulder and elbow injuries. Surprisingly, we initially expected that many pitches, rather than few pitches, would be a risk factor for injuries. Furthermore, we observed that a small number of daily pitches led to a 2.3-times greater risk of injuries and a 45-day earlier occurrence of injuries. No previous prospective study in high school pitchers has provided evidence that a small number of daily full-power pitches induces shoulder and elbow injuries in baseball players. This evidence may be useful for deciding the appropriate number of daily full-power pitches necessary to prevent shoulder and elbow injuries in high school baseball pitchers.

Takagishi et al.^3^, in a retrospective nationwide survey of junior high school baseball players, showed that 70 or more full-power pitches per day and 300 or more full-power pitches per week were significantly associated with the presence of elbow pain. In contrast, 70 or more full-power pitches per day were significantly associated with shoulder pain. Fleisig et al.^14^ have demonstrated that pitching more than 100 innings in 1 year was a significant risk factor for baseball-related arm injury. In particular, compared to players who pitched fewer than 100 innings per 1 year, players who pitched more than 100 innings in 1 year had approximately a 3.5-times higher occurrence of serious injury. Lyman et al.^2^ performed a prospective study among youth baseball pitchers and showed that throwing fewer than 300 or more than 600 pitches in games during the season was a risk factor for elbow pain. Although excessive game pitches lead to elbow pain by overuse^2,3,6^, pitchers exhibiting elbow pain early in the season are less likely to throw more than 300 pitches because of pain^2^; similarly, early elbow pain may be caused by inadequate arm conditioning^2^.

The abovementioned evidence is important for regulating excessive game pitches during the season. However, these studies did not investigate full-power daily pitches that may affect baseball-related arm injuries, as daily training sessions may be longer than an actual match. In addition, the number of cumulative daily pitches may be higher than that of cumulative game pitches. In this study, among high school baseball pitchers, we observed that pitchers who pitched fewer than 30 balls daily had approximately 2.3 times higher rate of injury than those who pitched 30 or more balls per day. Although surprising, not only an excessively high pitch volume but also an extremely low pitch volume could pose a risk of injury because continuous daily pitching is required to maintain physical condition in growing and maturing high school pitchers. However, as previous studies^3,14^ showed that a high pitch volume is one of the risk factors for a baseball-related arm injury, further studies will be required.

According to Pitch Smart USA Baseball (https://www.mlb.com/pitch-smart/pitching-guidelines), the maximum daily number of pitches is 95 and 105 among 15–16-year-old and 17–18-year-old pitchers, respectively, which corresponds to high school baseball pitchers. Additionally, the guideline establishes required rest recommendations: when pitchers aged ≥ 15 years pitch 1–30 balls daily, they do not require rest. Interestingly, the cut-off value of 30 pitches per day was similar to that derived in this study. Thus, around 30 pitches per day may be recommended as this pitch count limit requires no rest and is equally necessary for daily practice.

This study had some limitations. First, the data of other external load factors, such as the innings and training, and competition hours, were not collected in this study. These factors might be correlated with the number of daily pitches and could be confounding. Thus, this should be considered carefully in future studies. Second, the sample size was relatively small because the incidence of shoulder and elbow injuries was relatively low in high school baseball pitchers. However, post hoc power analysis showed that the statistical power of this study was 70.5%, indicating that the sample size was sufficient for testing the relationship between the number of full-power daily pitches and shoulder and elbow injury. Third, the relationship between the severity level and precise location of injuries and the baseball load was unclear; this is because we did not collect data on the severity level and precise location of injuries. Fourth, we asked the participants to memorize the number of daily pitches and record in questionnaires, and this might depend on the memorization ability of each participant. Although the participants were familiar with counting the full-power pitches as they have been educated continuously that self-management is necessary, individual differences in the memorization ability might affect the study result. Finally, the shoulder and elbow conditions (i.e. ROM and strength) just before injury were not collected. Although under-conditioning might affect a decrease in the number of pitches, all participants were active pitchers. In addition, the baseline conditions of participants, without ROM of elbow extension on the dominant side, in the S and L groups were not significantly different. These limitations should be investigated in future studies.

In conclusion, in high school baseball pitchers, a small number of daily pitches (< 30 pitches per day) led to 2.3 times greater risk of injuries and 45-day earlier occurrence of injuries than a large number of daily pitches (≥ 30 pitches per day). This evidence requires validation in future studies; however, it may form the basis for discussion and guidelines regarding the appropriate number of daily pitches required to prevent shoulder and elbow injuries in high school baseball pitchers.

## Methods

### Participants

We recruited 131 high school male baseball pitchers aged between 15 and 17 years following a preseason medical check-up. We explained the 150-day prospective study to all participants and invited them to join the study. Of all participants, 87 pitchers were interested in our study; subsequently, informed consent was obtained from the participants' parents. Finally, 87 pitchers were enrolled in this study.

Following the inclusion criteria of a previous study^10^, a pitcher was included if he (1) participated in preseason workouts as an active pitcher and (2) had no restrictions on pitching activities. In contrast, the exclusion criteria were (1) prior injuries (e.g., fracture) to the throwing arm; (2) the inability to throw or restricted pitching activity owing to a shoulder or elbow problem; or (3) performing daily muscle-specific rotator cuff training exercises or posterior capsule/sleeper stretches, other than during team exercises, because these activities were shown by a previous study^16^ to affect the incidence of baseball-related arm injury.

The Institutional Review Board of Gunma University Hospital (Identification number: 1003) approved this study. All methods were performed following relevant guidelines and regulations.

### Medical check-ups

As previously reported^10,16^, preseason medical check-ups were performed as a baseline medical examination. We aimed to evaluate the preseason condition of the participants’ shoulders and elbows. Participants’ hand dominance was not known to the examiners. Baseball experience, height, weight, shoulder and elbow ROM, and shoulder muscle strength were evaluated.

#### ROM measurements

As previously reported^10,16^, shoulder ROM of 90°, ABIR and HA, and elbow ROM of flexion and extension were measured by a certified orthopedic surgeon, using a digital protractor (iGaging, Los Angeles, CA, USA; minimum detectable unit = 0.1° ).

The intra-rater validity and reliability of ROM measurement using a digital protractor have been established in a previous study^10^. Passive ROM of ABIR was measured in the supine position with scapula stabilization by applying a posterior force to the coracoid process. To measure ABIR, a digital protractor was placed on the forearm. For passive ROM, HA was measured in the supine position with stabilization of the axillary border of the scapula. To measure HA, a digital protractor was placed on the humerus, and passive ROM of elbow flexion and extension were similarly measured in the supine position.

#### Strength measurements

PIR and PER strengths of the shoulder were measured using a PowerTrack II Commander hand-held dynamometer (J-Tech Medical, Salt Lake City, UT, USA; minimum detectable unit = 0.1 kg) by a certified orthopedic surgeon, as previously reported^7,10,16^. The intra-rater validity and reliability of hand-held dynamometers have been established in a previous study^10^. The median value of three repeated trials was recorded and analyzed. PER was measured in the prone position with the humerus in 90° abduction and the elbow in 90° flexion. The arm was set in a neutral position, and the examiner stabilized the humerus. The participant was asked to rotate their arms externally with maximum power against the dynamometer, placed on the dorsal side of the forearm, 5-cm proximal to the proximal wrist extension crease. PIR was measured similarly, except that the dynamometer was placed on the volar side of the forearm, 5-cm proximal to the proximal wrist flexion crease. The participant was asked to rotate the arm internally, with maximum power. The dominant to non-dominant ratios of PER and PIR were calculated for each participant.

### Injury tracking and season data collection

A shoulder or elbow injury was defined as any condition resulting in the pitcher being considered disabled for ≥ 8 days^10,16,17^. Other injuries that occurred via other mechanisms, such as trauma from falls, collisions with other players, sprains while running, or being hit by a ball, were not included in the statistical analyses. To determine when injuries occurred and record the exact number of daily full-power pitches, the participants were asked to memorize their daily full-power pitches and complete a self-recorded questionnaire, as soon as possible, regarding daily full-power pitches, the presence of shoulder and/or elbow pain, limitations to pitching caused by shoulder or elbow pain, the participation in games and daily practice, and the activeness as a pitcher (i.e. conversion of position to fielder). As previously reported^10,16^, to avoid recall bias and maintain the reliability of the daily questionnaire information, participants completed the questionnaire daily and sent the record to us every month. Furthermore, to confirm the completion of the daily questionnaires, we called the participants once or twice per month.

### Statistical analysis

We used the data collected until a shoulder and/or elbow injury occurred. We performed statistical analyses using SAS 9.4 (SAS Institute Inc., Cary, NC, USA). All tests were two-sided with a *P* < 0.05 significance level. ROC analysis and the Youden index were used to identify the cut-off point of the number of daily full-power pitches to determine the incidence of shoulder and elbow injuries. Subsequently, participants were categorized into groups comprising a small number of full-power pitches (S group) and a large number of full-power pitches (L group) using the detected cut-off value. The Mann–Whitney U test was used to evaluate differences between the groups. The Kaplan–Meier method was used to obtain time-to-event curves, and HRs were calculated for the incidence of injury, using Cox proportional hazard models. A log-rank test was performed to compare the survival distributions between the groups.

To calculate the number of participants required, we performed a priori statistical power analysis, which revealed that 39 participants would provide a statistical power of 80% at an α level of 0.05 with an HR of 2.7^10^, an accrual interval of 150 days, a follow-up interval of 150 days, and the median time to failure in the group, with the shortest time to failure of 50 days in the Kaplan–Meier analysis^18^. To evaluate the statistical power of this study, we performed a post hoc power analysis after data collection. Finally, we performed a sensitivity analysis to confirm the appropriateness of the detected cut-off value.

## Supplementary Information

Supplementary Figure S1.

Supplementary Information.

## Data Availability

The data supporting the findings of this study are available on request from the corresponding author H.S. The data are not publicly available because they contain information that could compromise the privacy of research participants.
