# Supplementary material for: A small number of daily pitches induces shoulder and elbow injuries among high school baseball pitchers: a prospective study
Source: Sci Rep. 2020 Dec 15;10:21955. doi: 10.1038/s41598-020-78957-0 (PMC7738486; doi:10.1038/s41598-020-78957-0)
Supplement: Supplementary file 1 — Supplementary Figure S1. [file 41598_2020_78957_MOESM1_ESM.docx]

**A small number of daily pitches induces shoulder and elbow injuries among high school baseball pitchers: A prospective study**

Hitoshi Shitara, Tsuyoshi Tajika, Takuro Kuboi, Tsuyoshi Ichinose, Tsuyoshi Sasaki, Noritaka Hamano, Takafumi Endo, Masataka Kamiyama, Atsushi Yamamoto, Tsutomu Kobayashi, Kenji Takagishi and Hirotaka Chikuda

**Figure Legends**

Figure S1. Receiver operating charcteristics (ROC) curve

ROC analysis showed that the cut-off value of the average numbers of daily full-power pitches of incidence of shoulder and elbow injuries was 30 pitches per day (P = 0.33, area under the curve [AOC] = 0.57).

**
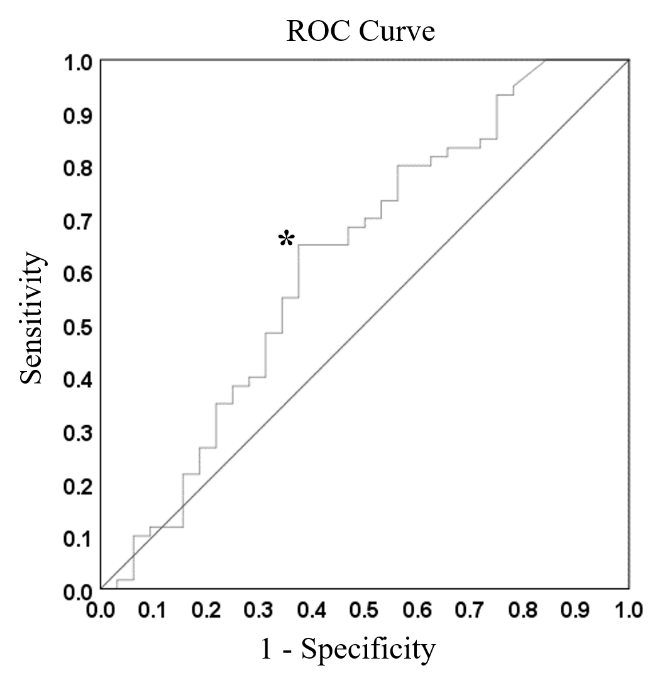
**

*****: Youden index
