# Supplementary material for: A small number of daily pitches induces shoulder and elbow injuries among high school baseball pitchers: a prospective study
Source: Sci Rep. 2020 Dec 15;10:21955. doi: 10.1038/s41598-020-78957-0 (PMC7738486; doi:10.1038/s41598-020-78957-0)
Supplement: Supplementary file 2 — Supplementary Information. [file 41598_2020_78957_MOESM2_ESM.docx]

**A small number of daily pitches induces shoulder and elbow injuries among high school baseball pitchers: A prospective study**

Hitoshi Shitara, Tsuyoshi Tajika, Takuro Kuboi, Tsuyoshi Ichinose, Tsuyoshi Sasaki, Noritaka Hamano, Takafumi Endo, Masataka Kamiyama, Atsushi Yamamoto, Tsutomu Kobayashi, Kenji Takagishi and Hirotaka Chikuda

**Sensitivity analysis of the cut-off value**

First, we set the shifted cut-off values by ± 5 (i.e. 25 and 35). Second, we performed the Kaplan-Meier analysis, Cox proportional hazard model analysis, and a log-rank test in the same manner as when a cut-off value of 30 pitches per day was used. Finally, we compared the results using the shifted cut-off values to that using a cut-off value of 30 to confirm the appropriateness of 30 pitches per day as a cut-off value in this study.

The results are shown below:

| Group | Total | Incidence of Injures | |  |
| --- | --- | --- | --- | --- |
|  | N | N (%) | HR (95% CI) | P-value |
| S-group (<25) | 26 | 12 (46.2) | 2.186 (1.086–4.399) | 0.024 |
| L-group (≥25) | 64 | 18 (28.1) | 1 |  |

| Group | Total | Incidence of Injures | |  |
| --- | --- | --- | --- | --- |
|  | N | N (%) | HR (95% CI) | P value |
| S-group (<35) | 47 | 19 (40.4) | 1.898 (0.915–3.937) | 0.078 |
| L-group (≥35) | 43 | 11 (25.6) | 1 |  |

HR; hazard ratio; CI, confidence interval.

A cut-off value of 30 pitches per day was superior in determining the incidence of throwing-related injury compared to the shifted cut-off values. Similarly, the sensitivity analysis supports the appropriateness of 30 pitches per day as the cut-off value in this study.
